# Lung Recruitment, Individualized PEEP, and Prone Position Ventilation for COVID-19-Associated Severe ARDS: A Single Center Observational Study

**DOI:** 10.3389/fmed.2020.603943

**Published:** 2021-01-22

**Authors:** Ling Sang, Xia Zheng, Zhanqi Zhao, Min Zhong, Li Jiang, Yongbo Huang, Xiaoqing Liu, Yimin Li, Dingyu Zhang

**Affiliations:** ^1^State Key Lab of Respiratory Diseases, Department of Critical Care Medicine, Guangzhou Institute of Respiratory Health, The First Affiliated Hospital of Guangzhou Medical University, Guangzhou, China; ^2^Department of Critical Care Medicine, The First Affiliated Hospital of Zhejiang University, Hangzhou, China; ^3^Department of Biomedical Engineering, Fourth Military Medical University, Xi'an, China; ^4^Institute of Technical Medicine, Furtwangen University, Villingen-Schwenningen, Germany; ^5^Department of Critical Care Medicine, Zhongshan Hospital, Fudan University, Shanghai, China; ^6^Department of Critical Care Medicine, Xuanwu Hospital, Capital Medical University, Beijing, China; ^7^Research Center for Translational Medicine, Wuhan Jinyintan Hospital, Wuhan, China; ^8^Joint Laboratory of Infectious Diseases and Health, Wuhan Institute of Virology and Wuhan Jinyintan Hospital, Chinese Academy of Sciences, Wuhan, China

**Keywords:** coronavirus disease 2019, acute respiratory distress syndrome, lung recruitability, PEEP titration, prone position ventilation

## Abstract

**Background:** Patients with coronavirus disease 2019 (COVID-19) may develop severe acute respiratory distress syndrome (ARDS). The aim of the study was to explore the lung recruitability, individualized positive end-expiratory pressure (PEEP), and prone position in COVID-19-associated severe ARDS.

**Methods:** Twenty patients who met the inclusion criteria were studied retrospectively (PaO_2_/FiO_2_ 68.0 ± 10.3 mmHg). The patients were ventilated under volume-controlled mode with tidal volume of 6 mL/kg predicted body weight. The lung recruitability was assessed *via* the improvement of PaO_2_, PaCO_2_, and static respiratory system compliance (C_stat_) from low to high PEEP (5–15 cmH_2_O). Patients were considered recruitable if two out of three parameters improved. Subsequently, PEEP was titrated according to the best C_stat_. The patients were turned to prone position for further 18–20 h.

**Results:** For recruitability assessment, average value of PaO_2_ was slightly improved at PEEP 15 cmH_2_O (68.0 ± 10.3 vs. 69.7 ± 7.9 mmHg, baseline vs. PEEP 15 cmH_2_O; *p* = 0.31). However, both PaCO_2_ and C_stat_ worsened (PaCO_2_: 72.5 ± 7.1 vs. 75.1 ± 9.0 mmHg; *p* < 0.01. C_stat_: 17.5 ± 3.5 vs. 16.6 ± 3.9 ml/cmH_2_O; *p* = 0.05). Only four patients (20%) were considered lung recruitable. Individually titrated PEEP was higher than the baseline PEEP (8.0 ± 2.1 cmH_2_O vs. 5 cmH_2_O, *p* < 0.001). After 18–20 h of prone positioning, investigated parameters were significantly improved compared to the baseline (PaO_2_: 82.4 ± 15.5 mmHg. PaCO_2_: 67.2 ± 6.4 mmHg. C_stat_: 20.6 ± 4.4 ml/cmH_2_O. All *p* < 0.001 vs. baseline).

**Conclusions:** Lung recruitability was very low in COVID-19-associated severe ARDS. Individually titrated PEEP and prone positioning might improve lung mechanics and blood gasses.

## Introduction

The rapid outbreak of coronavirus disease 2019 (COVID-19) has recently become a public health emergency of international concern (1). As of March 30, 2020, a total of 693,224 confirmed cases globally with 33,106 deaths (4.8%) had been reported by WHO (2). According to the result of a recent study, 16% of the COVID-19 patients developed to the severe cases, and 3.1% required invasive mechanical ventilation due to acute respiratory distress (ARDS) syndrome in China (3). Although the lung protective ventilation (LPV) strategy has been accepted worldwide (4), detailed clinical practices remain controversial due to the heterogeneity of ARDS. So far, the research revealing pathophysiology of COVID-19-associated ARDS is limited. The best approach of LPV is yet to be found. CT findings of COVID-19 patients suggested that opacities presented from focal unilateral to diffuse bilateral within 1–3 weeks (5, 6). A previous multicenter randomized controlled study suggested that patients with focal ARDS should receive low positive end-expiratory pressure (PEEP), prone position, and no recruitment maneuver (7). We hypothesized that even in a later stage of COVID-19, lung recruitability of severe ARDS was low, and the prone position could be beneficial. The aim of the present study was to examine our hypothesis in a retrospective cohort.

## Methods

### Design

This was a retrospective cohort study conducted in a 16-bed academic intensive care unit (ICU) at Jinyintan Hospital, which is one of the designated hospitals for COVID-19 patients in Wuhan, China. The authors Dr. Ling Sang and Dr. Xia Zheng were in charge of this ICU directly, according to the arrangement of the National Health Committee in the period of the COVID-19 outbreak. Local institutional research ethics board approved the study and the informed consent was waved due to the nature of the retrospective study.

### Patients

The consecutive laboratory-confirmed COVID-19 patients, who met the criteria of severe ARDS (8) and received invasive mechanical ventilation from Feb 8 to Feb 29 2020, were included in the study. Patients in early stage of COVID-19 (i.e., symptom onset <14 days) were not included. Further exclusion criteria included undrained pneumothorax, hemodynamic instability, obstructive lung disease, and other contraindications for high PEEP of 15 cmH_2_O or prone positioning.

### Measurement

Patients were paralyzed with propofol, midazolam, Remifentanil, and Cisatracurium and ventilated under volume-controlled mode with SV300 (Mindray, Shenzhen, China). To stabilize hemodynamics, continuous infusion was administrated, and the vital signs were closely monitored (patient monitoring systems from various manufactories). The assessment of lung recruitability and the prone-position efficacy were routinely performed according to our internal guideline for severe ARDS patients as soon as they were transferred to the ICU. In brief, the patients were first ventilated at the baseline with the following initial settings: tidal volume 6 mL/kg of predicted body weight; inspiratory time 0.9 s; PEEP of 5 mH_2_O; respiratory rate 25 breaths per min, and fraction of inspiratory oxygen (FiO_2_) 100%. After 5 min of the baseline period, PEEP was increased to 15 cmH_2_O for a further 5 min (the other ventilator settings remained unchanged). At the end of each PEEP phase, a 3-s end-inspiratory hold was performed. Various pressure values (peak pressure, P_peak_; plateau pressure, P_plat_) and the results of blood gas analysis (arterial oxygen partial pressure, PaO_2_ and arterial carbon dioxide partial pressure, PaCO_2_) were recorded. Driving pressure (P_driving_) was calculated as P_plat_ – PEEP. Static respiratory system compliance (C_stat_) was calculated as tidal volume divided by P_driving_. If two of the following three parameters improved—PaO_2_ (increase), PaCO_2_ (decrease), and C_stat_ (increase)—the patient was considered recruitable (9). Decremental PEEP trial with steps of 2 cmH_2_O and duration of 2 min was conducted subsequently to determine a proper PEEP based on the best C_stat_. Finally, patients were turned to prone position for 18–20 h with the ventilator settings unchanged. Before the patients were turned back to supine position, the lung mechanics and blood gases were again collected.

### Statistical Analysis

Data analysis was performed using MATLAB R2015a (The MathWorks Inc., Natick, USA). The Lilliefors test was used for normality testing. For normally distributed data, results were expressed as mean ± standard deviation (SD). Bland-Altman analysis was used to show the differences in the lung mechanics and blood gasses between different time points and baselines. Two-tailed paired-sample *t*-test was used to assess if the improvements of lung mechanics and blood gasses were significant. *P* < 0.05 was considered statistically significant. Bonferroni correction was used to adjust the *p*-value for multiple comparisons.

## Results

A total of 20 patients who met the criteria were included in the analysis. Baseline characteristics and clinical measures were summarized in Table 1. The PaO_2_/FiO_2_ values and C_stat_ of these patients were extremely low (68.0 ± 10.3 mmHg for PaO_2_/FiO_2_; 17.5 ± 3.5 ml/cmH_2_O for C_stat_).

**Table 1:** Baseline characteristics and clinical measures of the study patients.

**Pat. No**.	**Gender**	**Age (yr)**	**APACHE II**	**Tidal volume (ml)**	**PaO_**2**_/FiO_**2**_ (mmHg)**	**C_**stat**_ (ml/cmH_**2**_O)**
1	M	48	21	420	69	16.8
2	M	67	23	390	72	13.4
3	M	75	27	400	68	16.0
4	M	68	26	400	59	20.0
5	M	78	22	380	55	11.5
6	F	68	19	360	73	12.9
7	M	70	26	360	81	13.8
8	M	69	20	430	94	18.7
9	M	77	24	420	73	22.1
10	F	76	27	420	71	22.1
11	F	69	22	380	58	14.6
12	M	68	28	380	55	19.0
13	F	68	26	400	61	17.4
14	M	58	21	380	64	15.8
15	F	76	29	390	71	17.7
16	M	77	23	330	67	14.3
17	M	70	26	400	57	20.0
18	F	88	30	400	68	22.2
19	F	48	18	450	58	17.3
20	F	71	24	380	85	23.8
Mean M:F 12:8		69.5	24.1	393.5	68.0	17.5
SD		9.5	3.4	27.2	10.3	3.5

*Pat. No., patient number; M, male; F, female; APACHE, acute physiology and chronic health evaluation; PaO_2_/FiO_2_, ratio of arterial partial pressure of oxygen and fraction of inspired oxygen; C_stat_, static respiratory system compliance; SD, standard deviation*.

**Figure 1:**
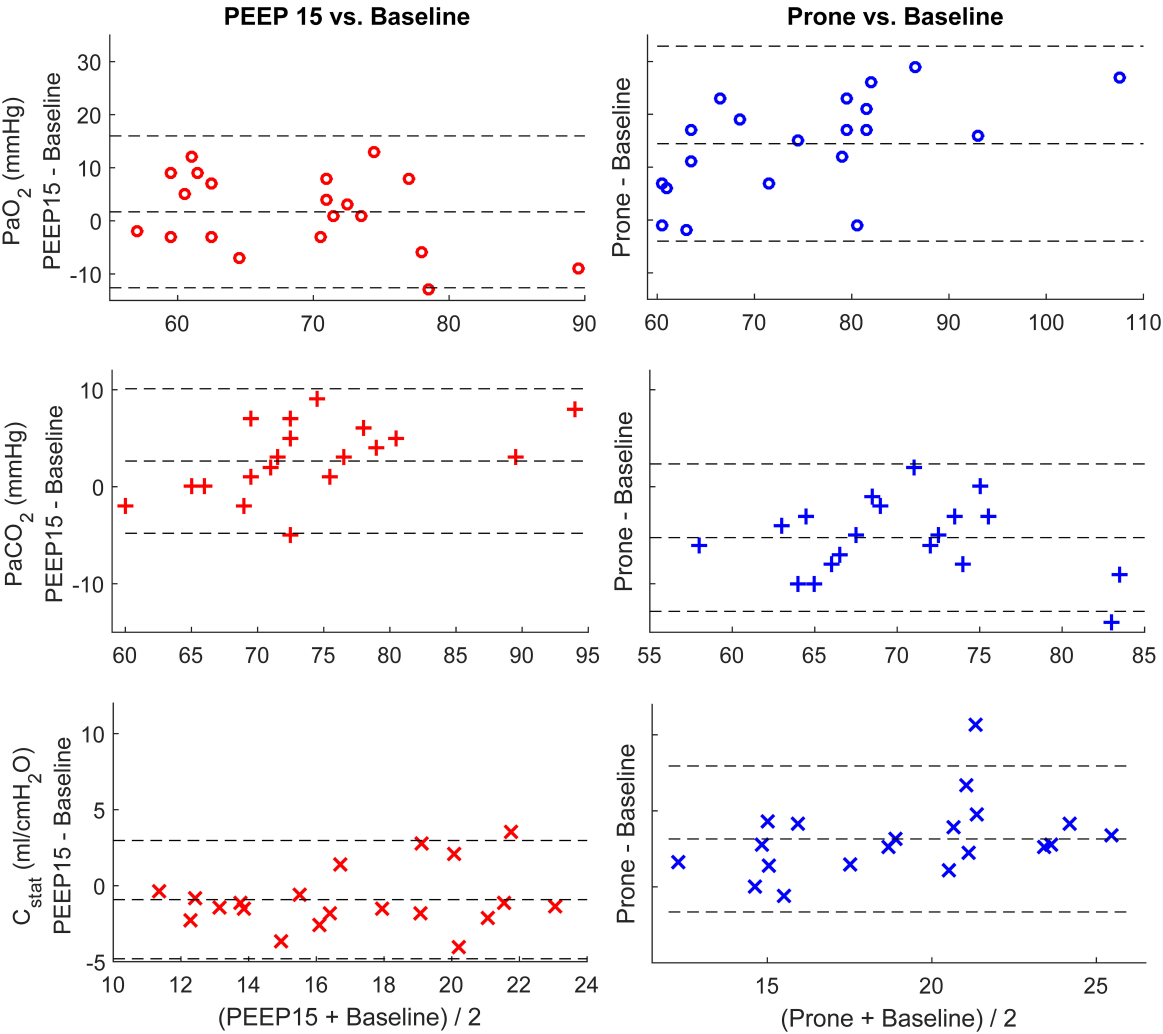
Bland-Altman plots comparing individual differences in PaO_2_, PaCO_2_, and C_stat_. Left column, comparing the parameters at PEEP of 15 cmH_2_O and baseline (PEEP of 5 cmH_2_O); right column, comparing the parameters after prone position and baseline. The dashed lines at the middle depict the mean values of the whole data set. The other two dashed lines represent mean ± 1.96 × standard deviation.

The changes of PaO_2_, PaCO_2_, and C_stat_ between a PEEP of 15 cmH_2_O and the baseline are plotted in Figure 1 left column. The mean and SD are also compared in Table 2. The average value of PaO_2_ was slightly improved. However, both PaCO_2_ and C_stat_ worsened at a PEEP of 15 cmH_2_O. The titrated PEEP after recruitability assessment was significantly higher than the baseline one (8.0 ± 2.1 cmH_2_O, *p* < 0.001) and lower than a PEEP of 15 cmH_2_O (*p* < 0.001). After 18–20 h in the prone positioning, all three parameters were significantly improved (Figure 1 right column; Table 2).

**Table 2:** Summary of the mean and SD of the investigated parameters at different time points.

**Parameters**	**Baseline**	**PEEP 15**	***P* vs. Baseline**	**After Prone**	***P* vs. Baseline**
PaO_2_ (mmHg)	68.0 ± 10.3	69.7 ± 7.9	0.31	82.4 ± 15.5	<0.001^*^
PaCO_2_ (mmHg)	72.5 ± 7.1	75.1 ± 9.0	<0.01^*^	67.2 ± 6.4	<0.001^*^
C_stat_ (ml/cmH_2_O)	17.5 ± 3.5	16.6 ± 3.9	0.05	20.6 ± 4.4	<0.001^*^

* *Indicates a statistically significant difference compared to the baseline*.

**Figure 2:**
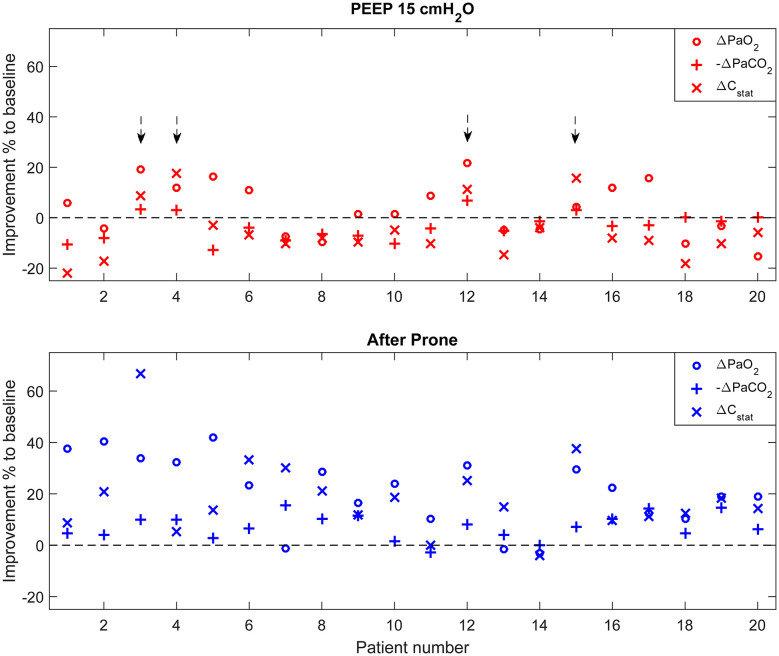
Individual improvement after PEEP increase and prone positioning compared to baseline. Parameter values were normalized to the corresponding values at the baseline. For PaCO_2_, improvement was defined as the negative change of PaCO_2_ compared to baseline higher than zero (–Δ PaCO_2_). Arrows (top) highlight the patients who were considered lung recruitable according to the criterion (improvement found in equal or more than two parameters).

Figure 2 summarizes individual improvement after PEEP increase and prone positioning. Four out of 20 patients (20%) were considered lung recruitable according to the criterion (Figure 2 top). On the other hand, 18 patients (90%) had improvements in more than two parameters after 18–20 h in the prone position (Figure 2 bottom).

## Discussion

In the present study, we have several findings regarding the patient cohort (COVID-19 patients with PaO_2_/FiO_2_ <100 mmHg): (1) low PaO_2_ and C_stat_, high PaCO_2_ despite of high respiratory rate (25 breaths per min); (2) the lung recruitability was low in most of the studied subjects (16 out of 20 were non-recruitable, 80%); (3) increased PaCO_2_ and decreased C_stat_ at PEEP of 15 cmH_2_O compared to baseline indicated that previously ventilated alveoli were already overdistended; and (4) individualized PEEP and prone position significantly improved PaO_2_, PaCO_2_, and C_stat_.

COVID-19 does not lead to a classical ARDS. A recent study suggested that COVID-19-associated ARDS has low recruitability (10). Knowing that the opacities in the patients' lungs would develop to diffuse bilateral within 1–3 weeks (5, 6), which might be more recruitable (7), we designed this retrospective study to analyze COVID-19 patients who had the onset of symptoms over 2 weeks. Although the methods of assessing lung recruitability were different to the previous study (10), the findings were comparable (80 vs. 83% patients poorly recruitable). We noticed that our subjects had high PaCO_2_, and therefore the respiratory rate was set to 25 breaths per min. However, despite such a high minute volume (9.8 ± 0.7 liter), PaCO_2_ still increased at a PEEP of 15 cmH_2_O, which indicated a dramatic increase of dead space. Combining the observation that C_stat_ was extremely low and decreased at a PEEP of 15 cmH_2_O, we speculated that a PEEP of 15 cmH_2_O was too high and introduced overdistension in opened lung alveoli. Driving pressure in our study subjects was relatively high, but given the remarkably poor blood gasses and lung mechanics, tidal volume could not be further reduced. Extracorporeal membrane oxygenation could be an option to protect the lung but, it was unavailable in this pandemic. Constantin and his colleagues have reported that recruitment maneuver would induce more overdistension in focal ARDS than in non-focal ARDS (11). It seems that although patients with COVID-19 developed to non-focal ARDS at a later stage, the impact of high pressure on lung tissues was similar to focal ARDS. Extreme caution should be given when conducting recruitment maneuver and applying high PEEP. A recent retrospective study showed that the average PEEP used for COVID-19 patients under invasive mechanical ventilation was 14 cmH_2_O in the Lombardy region of Italy (12). Considering the PaO_2_/FiO_2_ ratio [median (interquartile range): 160 mmHg (114–220 mmHg)] in the Italian cohort was much higher than our subjects, the applied PEEP might be too high in some of the patients. The worsening of blood gases may have a certain time delay after overdistension. A bedside tool such as electrical impedance tomography (13) could be considered to monitor the process closely.

In a recent letter to the editor, Gattinoni et al. have reported that prone positioning improved oxygenation in the COVID-19 patients they treated (14). They suspected that the improvement was mainly due to the redistribution of perfusion, which might not be the reason for our patient cohort, since C_stat_ of our subjects were much lower than theirs (17.5 ± 3.5 vs. 50.2 ± 14.3 ml/cmH_2_O). The improvement of the parameters we monitored (PaO_2_, PaCO_2_, and C_stat_) suggested that recruitment occurred in the prone position in our patients. Pan et al. found that the recruitability might be improved after the prone position, which was only demonstrated in 31% (13 out of 42) of their measurement events (10). Due to the nature of retrospective analysis, we could not distinguish the effect of individually titrated PEEP and prone positioning. An individualized moderate PEEP level could be helpful to recruit collapsed lung alveoli. Previous studies have proven that titrated PEEP could be more lung protective compared to a fixed PEEP (15). Besides, different PEEP titration methods may result in various PEEPs and lead to different outcomes (16). Nevertheless, our findings clearly supported the use of individualized PEEP and prone position in COVID-19-associated severe ARDS.

Our study has several limitations. The recruitability and the effect of PEEP and prone positioning were only routinely assessed once at the beginning of ICU admission to develop ventilation strategy. A prospective study should be performed to examine if the recruitability and the effect of PEEP and prone positioning would change over time at different disease stages. The number of study subjects was limited, and the study design was a single center retrospective. It would be interesting to know whether the severity would affect the patients' outcomes and recruitabilities. But with a limited number of subjects, it was impossible to divide the subjects into subgroups for further analysis. Nevertheless, with the shared experience, we hope that a corresponding lung-protective ventilation strategy could be developed for COVID-19-associated severe ARDS.

## Conclusions

Lung recruitability was very low in COVID-19-associated severe ARDS. Clinically used PEEP for classical ARDS could have induced overdistension. Individually titrated PEEP and prone positioning might improve lung mechanics and blood gasses.

## Data Availability Statement

The raw data supporting the conclusions of this article will be made available by the authors, without undue reservation.

## Ethics Statement

The studies involving human participants were reviewed and approved by research ethics board of Wuhan Jinyintan Hospital. Written informed consent for participation was not required for this study in accordance with the national legislation and the institutional requirements.

## Author Contributions

LS, XZ, XL, YL, and DZ contributed to the design of the conception and design of the study. LS, XZ, ZZ, MZ, and LJ were responsible for patient screening and enrollment. YH performed the statistical analysis. LS, XZ, YH, XL, YL, and DZ analyzed the data and wrote the manuscript. All authors contributed to interpretation of the data. All authors read and approved the final manuscript.

## Conflict of Interest

The authors declare that the research was conducted in the absence of any commercial or financial relationships that could be construed as a potential conflict of interest.

## Abbreviations

COVID-19: coronavirus disease 2019
ARDS: acute respiratory distress syndrome
PEEP: positive end-expiratory pressure
C_stat_: static respiratory system compliance
LPV: lung protective ventilation
ICU: intensive care units
FiO_2_: fraction of inspiratory oxygen
PaO_2_: arterial partial pressure of oxygen
PaCO_2_: arterial partial pressure of carbon dioxide
P_peak_: peak pressure
P_plat_: plateau pressure
P_driving_: Driving pressure
SD: standard deviation.
